# Haemodynamic outcomes during piston-based mechanical CPR with or without active decompression in a porcine model of cardiac arrest

**DOI:** 10.1186/s13049-018-0496-z

**Published:** 2018-04-24

**Authors:** Mikkel T. Steinberg, Jan-Aage Olsen, Morten Eriksen, Andres Neset, Per Andreas Norseng, Jo Kramer-Johansen, Bjarne Madsen Hardig, Lars Wik

**Affiliations:** 1 Institute of Clinical Medicine, University of Oslo, Oslo, Norway; 2 Norwegian National Advisory Unit on Prehospital Emergency Medicine, Oslo University Hospital, Oslo, Norway; 3 Department of Oncology, Oslo University Hospital, Oslo, Norway; 4 Institute of Experimental Medical Research, Oslo University Hospital, Oslo, Norway; 5 County Governor of Rogaland, Stavanger, Norway; 6 Physio-Control/Jolife AB, Ideon Science Park, Lund, Sweden

**Keywords:** Cardiac arrest, Active decompression, Experimental porcine model

## Abstract

**Background:**

Experimental active compression-decompression (ACD) CPR is associated with increased haemodynamic outcomes compared to standard mechanical chest compressions. Since no clinically available mechanical chest compression device is capable of ACD-CPR, we modified the LUCAS 2 (Physio-Control, Lund, Sweden) to deliver ACD-CPR, hypothesising it would improve haemodynamic outcomes compared with standard LUCAS CPR on pigs with cardiac arrest.

**Methods:**

The modified LUCAS delivering 5 cm compressions with or without 2 cm active decompression above anatomical chest level was studied in a randomized crossover design on 19 Norwegian domestic pigs. VF was electrically induced and untreated for 2 min. Each pig received ACD-CPR and standard mechanical CPR in three 180-s. phases. We measured aortic, right atrial, coronary perfusion, intracranial and oesophageal pressure, cerebral and carotid blood flow and cardiac output. Two-sided paired samples t-test was used for continuous parametric data and Wilcoxon test for non-parametric data. *P* < 0.05 was considered significant.

**Results:**

Due to injuries/device failure, the experimental protocol was completed in nine of 19 pigs. Cardiac output (l/min, median, (25, 75-percentiles): 1.5 (1.1, 1.7) vs. 1.1 (0.8, 1.5), *p* < 0.01), cerebral blood flow (AU, 297 vs. 253, mean difference: 44, 95% CI; 14–74, *p* = 0.01), and carotid blood flow (l/min, median, (25, 75-percentiles): 97 (70, 106) vs. 83 (57, 94), *p* < 0.01) were higher during ACD-CPR compared to standard mechanical CPR. Coronary perfusion pressure (CPP) trended towards higher in end decompression phase.

**Conclusion:**

Cardiac output and brain blood flow improved with mechanical ACD-CPR and CPP trended towards higher during end-diastole compared to standard LUCAS CPR.

## Background

Mechanical chest compressions during cardiac arrest have improved hemodynamic variables in porcine and human studies and been documented to be safe with equal survival rates to high quality manual chest compressions during OHCA [1–6]. The use of active compression-decompression CPR (ACD-CPR), with active decompression to a higher level than the normal anatomical level, has showed promising results compared to standard chest compressions in both animals and humans [7–17]. These studies utilized either large mechanical power driven customized devices or a handheld device (CardioPump) to deliver ACD-CPR [7–17]. The mechanical devices used in the animal lab were impossible to bring into the field, and use of the handheld device did not deliver the same level of standardization and continuity as mechanical devices, resulting in lower fractions of ACD-CPR adhering to guidelines [18, 19]. The handheld device has been studied extensively, both alone and combined with an impedance threshold device (ITD). Systematic reviews conclude that neither manual ACD-CPR nor the ITD-device during manual CPR improve long time survival. However, Aufderheide et al. demonstrated increased survival when combining the two techniques [20–23].

No commercially available automatic mechanical chest compression device has so far been able to perform ACD-CPR. Such a device would be of both academic and clinical interest since a mechanical device can enable consistent high quality ACD-CPR independent of rescuer fatigue. We hypothesized that the commercially available piston-based battery/mains powered mechanical chest compression device LUCAS 2 (Physio-Control/Jolife AB, Lund Sweden) modified to deliver ACD-CPR, would improve hemodynamic parameters during cardiac arrest in pigs compared with standard mechanical compressions delivered by LUCAS 2.

## Methods

### Study design

This study compared mechanical CPR during ventricular fibrillation (VF) with piston-based chest compressions (LUCAS 2) with and without active decompression to 2 cm above normal anatomical level. Each pig served as its own control in cross over design. After surgery and preparation, but before induction of VF, pigs were randomized by drawing one of 19 envelopes where the sequence of the CPR techniques was written. A balanced design was achieved with each CPR technique performed once or twice on each pig the same number of times (intervention-control-intervention, or control-intervention-control).

The experiments were carried out in accordance with “Regulations on Animal Experimentation” under The Norwegian Animal Welfare Authority Act and approved by Norwegian Animal Research Authority (FOTS-ID 4931).

### Animal preparation and instrumentation

Healthy Norwegian domestic pigs of both genders fasted eight hours prior to the experiment, but had access to water. Anaesthesia was induced with i.m. ketamine 30 mg/kg, atropine 1 mg and morphine 10 mg. A venous catheter was placed in the ear for infusion of Ringer acetate 30 ml/kg/h and induction of anaesthesia with fentanyl 10 microgram/kg and propofol 2 mg/kg i.v. Anaesthesia was maintained by infusion of fentanyl (3–10 microgram/kg/min) and propofol 2–10 mg/kg/h guided by hemodynamic response and need. The pig was intubated and ventilated with Datex-Ohmeda S5 ventilator (FIO_2_ 0.3, respiration rate (RR) 16/min and tidal volume (TV) 15 ml/kg) targeted to expired end tidal carbon dioxide (EtCO_2_) of 5.0 ± 0.5 kPa measured by Cosmo plus (Novametrix Medical systems, Wallingford, CT USA). Mean arterial pressure (MAP) was maintained between 65 and 90 mmHg with the use of Ringer Acetate if needed.

The pig was then placed on its back on a U-shaped bed and all limbs were fastened and the head fixated. The temperature was measured by a urine catheter placed via cystotomy and maintained at 38.0 ± 0.5° C with the help of a heating/cooling mattress (Artic Sun, Medivance, Louisville, CO, USA).

Defibrillation pads placed in the upper right quadrant of the chest and lateral to columna on the left side of the chest were connected to a LIFEPAK 12 Monitor/Defibrillator (Physio-Control, Redmond, WA, US).

The common and external carotid arteries were dissected. A Doppler flow meter probe (model 3SB880, Transonic Systems Inc., Ithaca, NY, USA) was placed on the right common carotid artery, and the external carotid artery was ligated. Two 7F micro-tip pressure transducer catheters (Model SPC 470, Millar Instruments, Houston, TX, USA) were placed, one through the right femoral artery up to the aortic arch and for continuous arterial pressures measurements (SAP = systolic aortic pressure, MAP = mean aortic pressure, DAP = diastolic aortic pressure), the second catheter was placed through the left external jugular vein to the right atrium for continuous pressure measurements (SRAP = systolic right atrial pressure, MRAP = mean right atrial pressure, DRAP = diastolic right atrial pressure). A 7.5F Swan-Ganz catheter (Edwards Lifesciences, Irvin, CA, USA) was placed in the pulmonary artery via the right femoral vein for thermodilution cardiac output and wedge pressure measurements. Another 7.5F Swan-Ganz catheter was placed in the right atrium through the left femoral vein, and a fluid filled polyethylene catheter was placed in aorta through the left femoral artery. These catheters were used for blood gases.

Oesophageal pressure was measured at the level of the heart using a cylindrical shaped rubber balloon (length 5 cm, perimeter 3.4 cm) containing air, glued to an open ended 7-F stiff catheter with multiple side holes, attached to a pressure transducer.

Craniotomy and duratomy were performed 10 mm anterior of the coronary suture and 15 mm to the left of the lateral part of the sagittal suture for a laser Doppler flowmeter probe (Modell 407, Perimed AB, Stockholm, Sweden) on the surface of cerebral cortex.

The skin over sternum was dissected and an oval shaped metal plate (12 × 6 cm) secured to the sternum with 6–8 screws. A removable metal pin enabled fastening the modified LUCAS 2 device piston to the metal plate in order to achieve active decompression and pulling/lifting of the sternum when placed, and standard chest compressions when removed. The pin could be removed or inserted in 4–5 s. The LUCAS 2 device used in present study was only physically modified by removing the suction cup, all other modification allowing ACD-CPR with 2 cm of additional decompression were accomplished by software alteration carried out by Physio-Control/Jolife AB, Lund, Sweden. Two cm of decompression was chosen because this amount of decompression combined with 5 cm of compression yielded best hemodynamic results in an earlier study with similar design [8].

### Monitored variables

The following variables were monitored and continuously measured during the interventions: Systemic arterial pressure, right atrial pressure, intrathoracic pressure (oesophagus), cerebral blood flow (laser Doppler), carotid artery flow. Coronary perfusion pressure (CPP) was calculated as the difference between aortic pressure and right atrial pressure. In addition, the following variables were measured at specific time points during the experiment: cardiac output (CO), arterial and central venous blood gases (ABG and CVBG), EtCO_2_ and bladder temperature.

All continuously measured pressure and flow signals were conditioned with Gould Transducer amplifiers in a Gould 6600 chassis (Gould Electronics) and sampled with a PC data acquisition system (NI SCXI-1000, NI PCI-6036E, National Instruments Company, Austin, TX, USA) with VI Logger software (National Instruments Company, Austin, TX, USA) and broken down to a sampling frequency of 100 per chest compression cycle.

### Experimental protocol (Fig. 1)

We registered baseline measurements of pressures, flow, CO and EtCO_2_ after instrumentation and stabilization before induction of VF. A transcardial current (0.9 V DC) induced VF, which was verified by ECG and disappearance of pressures. At the same time anaesthesia, heating, i.v. fluids and ventilations were discontinued. No drugs were given during the three experimental phases. This non-circulatory state was continued for 2 min after VF induction. The chest wall was then «primed» for 30 s. with the mechanical chest compression device with 3 cm compression depth and a frequency of 102 ± 2/min. This was done in order to adjust the base level for the chest compression depth due to initial changes in chest configuration caused by chest compressions. Phase 1 started after the chest was “primed”. Pressures, flow and EtCO_2_ were measured continuously, cardiac output was measured at 60 and 150 s and blood gases (ABG, CVBG) 150 s into phase 1. The pigs were manually ventilated (FiO_2_ 1.0) by a person blinded for the ETCO_2_ value with a Laerdal bag connected to the endotracheal tube, 10–15 pr. min between chest compressions and a tidal volume of approximately 400–500 ml.

**Fig. 1:**
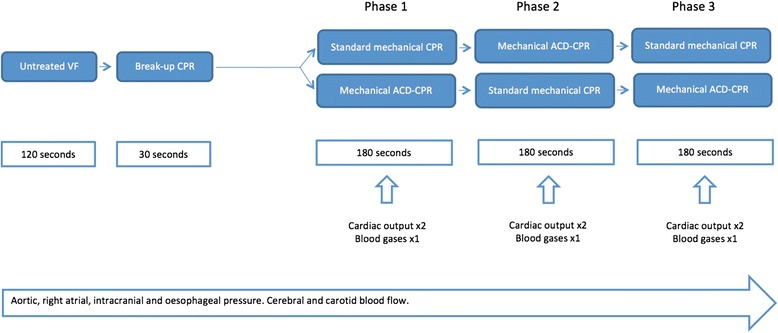
Illustration of the experimental protocol

#### Phase 1

The piston was adjusted in order to touch the metal plate on the chest and the first 180 s phase of CPR was started. Mechanical chest compressions were delivered at a 50/50 compression/decompression phase duty cycle, a rate of 102 ± 2/min and depth of 53 mm ± 2 mm (control) or with the same depth and rate in addition to active decompression to 2 cm above normal anatomical chest level (intervention).

#### Phase 2

Active decompression was added or withdrawn based on what was performed in phase 1.

#### Phase 3

Active decompression was added or withdrawn again, and the same method (control or intervention) carried out as in phase 1.

After phase three the pigs received an overdose of propofol and 20 ml 1 M KCl, and CPR was continued for 1 min. The experiment was finished when there was no pressure or flow generating cardiac activity. The chest wall and abdomen were opened in order to detect injuries and verify correct placement of the catheters.

### Power analysis

We know from previous studies during optimally performed mechanical chest compressions (50/50 compression/decompression cycle, depth 4 cm, rate 100/min) during VF in a porcine model that CPP is approximately 15–25 mmHg with standard deviation (SD) of 4.

Power analysis performed in Sample Power shows that with crossover and paired analysis 10 pigs were needed in order to demonstrate a CPP difference of 10 mmHg with the power of 0.99 and alfa 0.05. The smallest difference in CPP to be documented with 10 pigs, power of 0.9 and SD 4, is 4.5 mmHg.

For the secondary endpoint carotid artery flow as percentage of basal flow, analysis indicated a power of 0.80 for a difference of 0.20 (absolute change in percentage flow) with SD 0.20 with 10 experiments.

### Statistical analysis

We compared intervals during continuous mechanical chest compressions with or without active decompression. Two-sided paired samples t-test was used for continuous parametric data and Wilcoxon test for non-parametric data. *P* < 0.05 was considered significant. Analyses were performed using IBM SPSS version 23/24 (IBM Corp. Armonk, NY, USA). Primary endpoint was CPP and secondary endpoints were cerebral blood flow and other haemodynamic parameters.

## Results

### Haemodynamic results

A total of 19 pigs (34.0 ± 3.3 kg, 20.2 ± 0.9 cm AP chest diameter) were used in the present study, whereof 10 were excluded due to device failure or injury during the experiment. Among the nine included pigs, all aspects of the experimental protocol were concluded in eight. The experimental protocol had to be cancelled 45 s into phase 2 in one pig because of chest compression device failure. Data from both phase 1 and 2 in this pig were included in the analysis. The order of the three experimental phases was equally distributed among the remaining eight pigs. Descriptive characteristics and baseline values are presented in Table 1.

**Table 1:** Pig characteristic and pre-VF basal haemodynamic values

*N* = 9	Mean ± SD or Median & quartiles
Weight (kg)	34 ± 3.3
Anterior-Posterior chest-diameter (cm)	20 ± 0.9
Temperature (Celsius)	38 ± 0.6
Cardiac Output (l/min)	3.6 (3.1, 4.5)
EtCO_2_ (kPa)	5.4 ± 0.8
Aortic pressure (mmHg)	87 ± 6.9
Right atrial pressure (mmHg)	6.9 (6.4, 11)
Coronary perfusion pressure (mmHg)	77 (69, 84)
Intracranial pressure (mmHg)	14 ± 4.4
Oesophageal pressure (mmHg)	40 ± 38
Cerebral flow (AU)	452 ± 182
Carotid flow (ml/min)	181 ± 18
pH - Arterial blood gas	7.4 ± 0.2
pCO_2_ - Arterial blood gas (kPa)	6.0 ± 0.6
pO_2_ - Arterial blood gas (kPa)	10.8 ± 1.3
pH - Venous blood gas	7.3 ± 0.02
pCO_2_ - Venous blood gas (kPa)	7.3 ± 0.8
pO_2_ - Venous blood gas (kPa)	4.4 ± 0.6

There were no significant differences in mean aortic, right atrial, oesophageal or intracranial pressures, EtCO_2_, arterial or venous blood gases between ACD-CPR and standard mechanical CPR. Cardiac output, cerebral and carotid blood flows were significantly higher during ACP-CPR (Table 2).

**Table 2:** Comparison between standard mechanical CPR and ACD CPR

	Standard CPR mean ± SD	ACD-CPR mean ± SD	Mean difference (95% CI)	Standard CPR median (quartiles)	ACD-CPR median (quartiles)	*p*-value
Blood pressures (mmHg)						
Aortic pressure				55 (51, 70)	60 (51, 70)	0.86
Right atrial pressure	54 ± 16	55 ± 16	1.0 (− 4.0, 6.0)			0.66
Coronary perfusion pressure	2.2 ± 22	0.6 ± 20	− 2.7 (− 10, 4.7)			0.42
Oesophageal pressure	57 ± 38	60 ± 39	2.6 (− 2.6, 7.8)			0.28
Intracranial pressure	24 ± 6.4	23 ± 6.7	−0.7 (− 1.6, 0.1)			0.09
Blood flow						
Cerebral flow (AU)	253 ± 268	297 ± 264	44 (14, 74)			0.01
Carotid artery flow (ml/min)				83 (57, 94)	97 (70, 106)	< 0.01
Manual measurements						
Cardiac output (l/min)				1.1 (0.8, 1.5)	1.5 (1.1, 1.7)	< 0.01
End tidal CO_2_ (kPa)	2.2 ± 0.8	2.5 ± 1.0	0.3 (− 0.1, 0.7)			0.15
Blood gases (kPa)						
pH - Arterial				7.3 (7.2, 7.4)	7.3 (7.2, 7.4)	0.33
pCO_2_ - Arterial				5.6 (4.9, 8.0)	5.4 (4.4, 8.1)	0.89
pO_2_ - Arterial	22 ± 20	25 ± 21	2.5 (−6.2, 11)			0.52
pH - Venous				7.1 (7.1, 7.2)	7.2 (7,1, 7.2)	0.09
pCO_2_ - Venous	11 ± 1.9	11 ± 1.9	−0.3 (− 0.5, 0.0)			0.06
pO_2_ - Venous				3.1 (3.0, 3.6)	3.3 (3.1, 3.6)	0.11

**Fig. 2:**
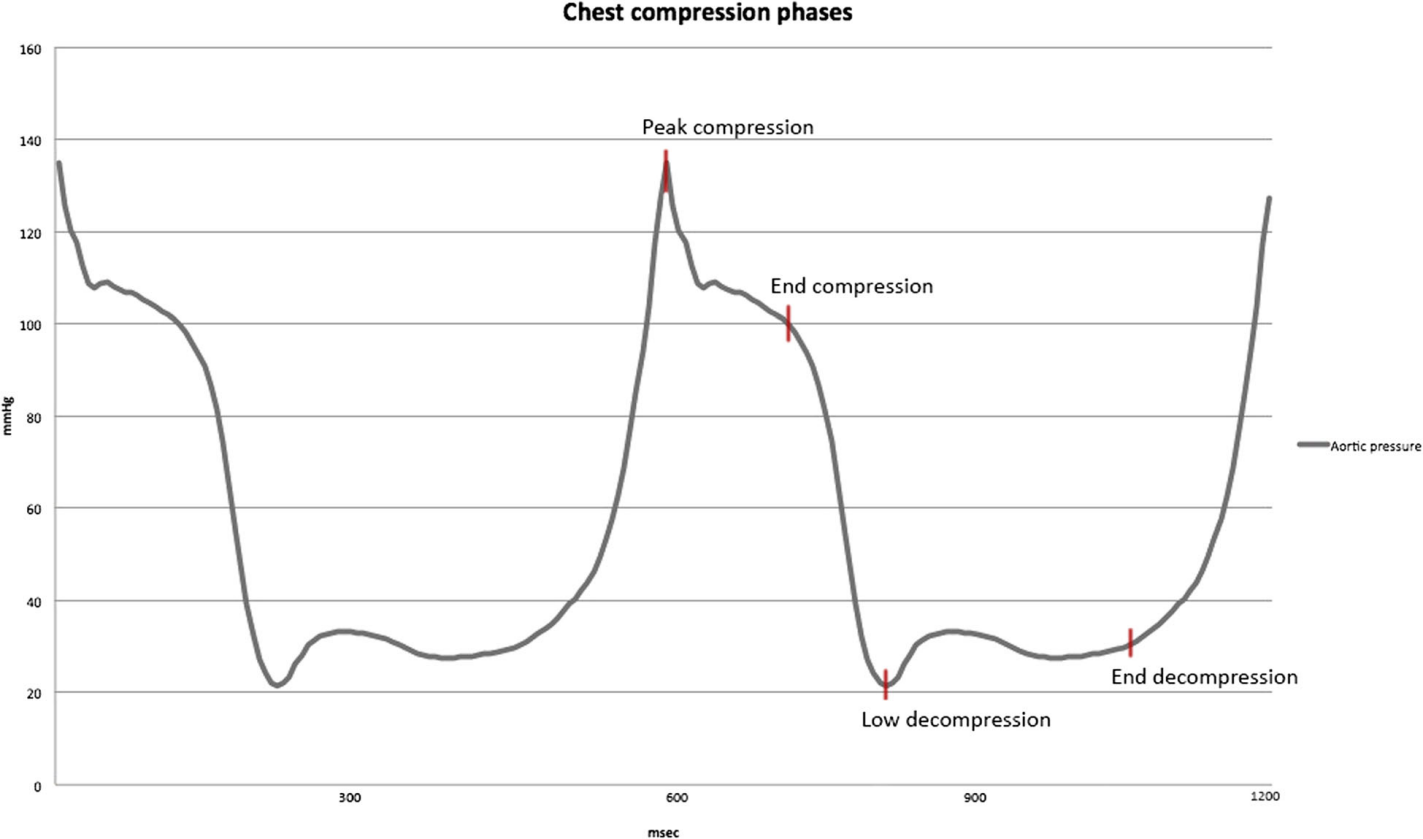
Demonstrates a pressure curve (aortic pressure in this example) and our definitions of the different phases of the chest compression cycle. Chest compression cycle phases was determined based on aortic pressure curves. y-axis = pressure (mmHg), x-axis = time

**Fig. 3:**
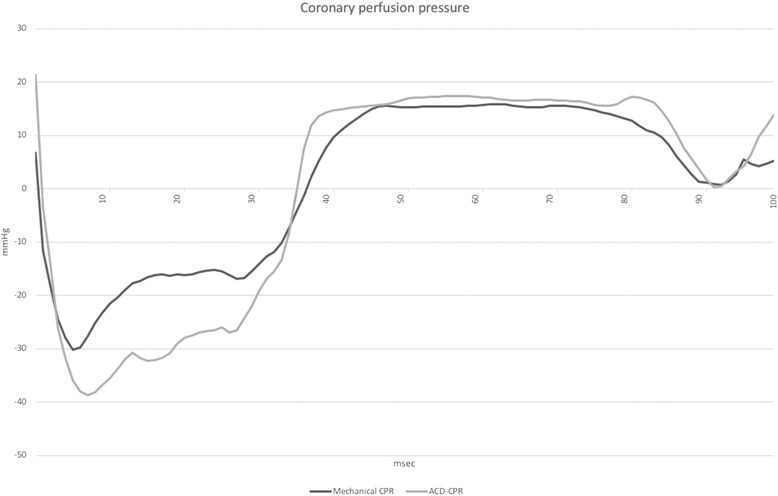
Average pressure curves for both standard mechanical CPR and ACD-CPR demonstrating coronary perfusion pressure

When analysing the different phases of the CPR cycle (Figs. 2, 3 and Table 3), aortic pressure was significantly higher in the peak compression phase during ACD-CPR. ICP was significantly lower in the end decompression phase during ACD-CPR. CPP trended towards higher values during ACD-CPR in the late decompression phase (*p* = 0.06). Cerebral blood flow was significantly higher during ACD-CPR during all phases of the CPR cycle, while carotid artery flow did not show any significant differences in any specific CPR cycle phase.

**Table 3:** Standard mechanical CPR compared with ACD CPR during different CPR cycle phases

	Standard CPR mean ± SD	ACD-CPR mean ± SD	Mean difference (95% CI)	Standard CPR median (quartiles)	ACD-CPR median (quartiles)	*p*-value
Blood pressures (mmHg)						
Aortic pressure						
Peak compression	180 ± 78	196 ± 79	16 (1.1, 30)			0.04
End compression	73 ± 65	68 ± 85	−4.9 (−29, 19)			0.65
Low decompression	10 ± 11	8.7 ± 13	−1.4 (−8.6, 5.8)			0.66
End decompression	28 ± 10	30 ± 9.1	2.0 (− 1.1, 5.2)			0.18
Right atrial pressure						
Peak compression	172 ± 71	180 ± 75	7.8 (− 17, 33)			0.49
End compression	91 ± 31	97 ± 32	6.3 (−3.2, 16)			0.16
Low decompression				7.7 (5.5, 22)	13 (5.6, 21)	0.59
End decompression				11 (11, 18)	11 (9.4, 17)	0.11
Coronary perfusion pressure						
Peak compression	8.3 ± 39	16 ± 44	7.8 (−9.8, 25)			0.34
End compression				−21 (−39, 6.0)	−19 (− 39, 6.5)	0.59
Low decompression	−1.2 ± 14	−6.2 ± 17	−4.9 (− 11, 1.4)			0.11
End decompression	12 ± 11	16 ± 9.4	4.3 (−0.2, 8.7)			0.06
Intracranial pressure						
Peak compression				38 (28, 44)	41 (24, 45)	0.37
End compression	35 ± 10	35 ± 12	−0.2 (−4.2, 3.8)			0.91
Low decompression	23 ± 8.3	24 ± 6.7	1.4 (−2.1, 4.8)			0.39
End decompression	15 ± 4.9	14 ± 5.2	−0.7 (−1.2, − 0.1)			0.03
Oesophageal pressure						
Peak compression	106 ± 83	118 ± 94	13 (−17, 42)			0.35
End compression	76 ± 48	84 ± 51	7.4 (−3.3, 18)			0.15
Low decompression	40 ± 37	40 ± 34.	−0.4 (−12, 11)			0.94
End decompression	39 ± 29	39 ± 32	0.0 (−7.8, 7.7)			0.99
Blood flow						
Cerebral flow (AU)						
Peak compression	248 ± 268	285 ± 261	38 (9.8, 66)			0.01
End compression	244 ± 255	278 ± 251	34 (8.7, 60)			0.02
Low decompression	250 ± 257	288 ± 254	38 (5.3, 70)			0.03
End decompression	266 ± 283	316 ± 283	51 (18, 82)			< 0.01
Carotid artery flow (ml/min)						
Peak compression	288 ± 93	311 ± 106	23 (−16, 62)			0.21
End compression				159 (59, 321)	176 (100, 223)	0.17
Low decompression				7.7 (−99, 51)	17 (−47, 97)	0.37
End decompression				22 (−19, 98)	38 (−7.7, 117)	0.09

### Injuries

Experimental/device failure and injuries during instrumentation or early experimental phase warranted the additional use of ten pigs in order to finish the study. The reasons for exclusion were as follows: A sternum fixation screw punctured the heart [*n* = 1]. Sternal fracture and puncture of the right atrium [*n* = 1]. Loosening of sternal screws and plate [*n* = 2] or breaking of sternal plate [*n* = 1]. Loss of cardiac output-values and/or large thoracic bleeding [*n* = 3]. Compression device failure [*n* = 2].

## Discussion

Our findings of increased cardiac output and cerebral blood flow during ACD-CPR are supported by earlier studies on dogs by Cohen et al. and Chang et al. [9, 12] As in most other studies they used a manual handheld device deliver ACD-CPR. Lindner et al. were the first to document higher cerebral blood flow during ACD-CPR when using piston based mechanical CPR in both ACD and control pigs [10].

No differences in mean pressures were demonstrated in present study, but aortic pressure was higher during peak compression with ACD-CPR, and CPP trended to be higher during end decompression with ACD-CPR (Fig. 2). Both these results are similar to what Wik et al. demonstrated in 1996 [8]. They also demonstrate lower oesophageal pressures during the end decompression phase with ACD-CPR, in addition to higher right atrial pressures during the peak and end compression phase of ACD-CPR. The present absolute values were similar, but we were unable to demonstrate significant differences. The data from Wik et al. were manually extracted based on printed pressure curves. Our data were collected by a real time data acquisition system and presented generally higher both aortic and right atrial pressures. Both studies found negative CPP values during what is equivalent to systolic parts of the chest compression cycle. This supports that CPP and thereby myocardial perfusion only takes place in the diastolic phase of mechanical ACD-CPR. CPP is not a major determinant of myocardial blood flow within the physiological range of arterial blood pressure. Myocardial perfusion is however directly related to CPP in low-pressure scenarios such as CPR [24]. CPR-induced high intrathoracic pressure in the compression phase has low impact on myocardial perfusion because this pressure is also applied to the right side of the heart, thus not generating the arteriovenous pressure difference needed for coronary perfusion [25]. A higher aortic pressure in the decompression phase combined with a reduction in right atrial pressure is therefore the key to achieving increased myocardial perfusion during CPR. In the present study there was only a not significant trend (*p* = 0.06) towards higher CPP in the end decompression phase. We did not find an additional decrease in oesophageal pressure as a proxy for reduced intrathoracic pressure during ACD-CPR. One could speculate that the possibility for a further reduction in right atrial pressure could be achieved by combining ACD-CPR with an ITD, as demonstrated by Aufderheide et al. [23] Langhelle et al. also demonstrated a significant decrease in right atrial pressure with ACD-CPR, both with and without ITD. The mean decrease was greater for ACD-CPR alone, but ACD-CPR and ITD combined resulted in a greater decrease in the early decompression phase. These differences were only statistical significant when compared with manual CPR, not when ACD with or without ITD were compared [26].

As already mentioned, both present and earlier studies indicate that the diastolic/decompression phase of the chest compression cycle is the period of coronary perfusion. Current and earlier papers have presented several points of measurement during the diastolic/decompression phase in order to demonstrate the change in pressure throughout the chest compression cycle [7, 8, 26].

In addition to pressures we also measured flow during the different phases of the chest compression cycle. Our data shows higher mean cerebral blood flow during ACD-CPR, with significantly higher blood flow during all chest compression phases. Carotid artery flow did also show higher mean values in the ACD group, however no chest compression phase demonstrated significantly higher values. Similar hemodynamic benefits have been found in humans with ACD-CPR, but not improved short or long-term survival [13–17, 21]. In a similar study design Langhelle et al. did not demonstrate differences in brain blood flow for ACD-CPR with or without ITD vs. standard CPR. They found increased coronary flow for ACD-CPR both with and without an ITD vs. standard CPR, but no significant difference between ACD-CPR with vs. without ITD [26].

There were no differences in blood gases between ACD-CPR and standard mechanical CPR, and there was no hyperventilation with potential impact on cerebral blood flow.

All clinical studies included in earlier systematic reviews of ACD-CPR utilized a handheld suction-based device to deliver ACD-CPR [20–22]. This device is reported to require more energy than regular manual CPR, and it has been documented that CPR quality suffers with significantly lower compression rate, depth and duration, in addition to inadequate decompression force compared to both regular manual and mechanical CPR [18, 19, 27]. This could partly explain why hemodynamic benefits of ACD-CPR in experimental studies failed to result in better clinical outcomes.

### Limitations

The results from this experimental porcine study are not directly transferable to clinical cardiac arrest. A high number of pigs were excluded from the trial because of injuries and failure during instrumentation and the experimental phase. The mechanical chest compression device was fastened to the sternum with screws on the pigs in present study. The clinical version of the device uses a suction cup to adhere to the human chest. Injuries during instrumentation would therefore not be applicable to the clinical setting of cardiac arrest. Among the ten exclusions, four were related to sternal plate/screw failure and two to machine failure. The remaining four pigs had severe injuries early in the experimental phase. These injuries were mainly due to rupture of large vessels in/out of the heart, heart tamponade, punctured right atrium, in addition to one episode of sternal fracture. We hypothesise that these injuries may have been a result of how the chest compression piston was fastened to a plate screwed directly on to the pig’s sternum, resulting in a very direct transfer of forces. The pull on this plate would be especially large during the transition between compression and decompression, and a suction cup would absorb a lot of this energy. The combination of such large drag forces and the fact that a pig’s pericardium adheres directly to the inside of the sternum could explain the injuries during the experimental phases of present study [28]. Future studies should take this into account and consider the use of a suction cup modified to a pig’s chest. We have no way to analyse if there were significant differences in injuries between the ACD-CPR and standard CPR as each pig was its own control.

We cannot rule out a carry-over effect from control to intervention or vice versa because of the cross-over nature of the study design. The three-phase design does take this into account and should at least partly make this kind of bias less likely by repeating the first modality.

We cannot rule out that the surgical preparation of the pig with dissection of the carotid arteries and catheter placement through jugular veins could alter the blood flow entering and exiting the brain.

No studies have to our knowledge validated the use of Swan Ganz catheters for measuring cardiac output during cardiac arrest. They are widely used for measuring pulmonary pressure in both pulmonary, cardiac and resuscitation research. Correct placement of all catheters was confirmed after completion of the experiment by autopsy.

The experimental protocol included two minutes of untreated VF. This is shorter than in most clinical situations, particularly for unwitnessed cardiac arrest or when bystander CPR is initiated after telephone instructions. The results are therefore not directly transferable.

An ITD was not included in the study although such devices have demonstrated both haemodynamic and clinical benefits earlier. We wanted to study the effect of mechanical active decompression alone in order to study the outcome of one intervention at a time. Including an ITD could be a natural next step.

## Conclusion

ACD-CPR delivered by a modified clinically used mechanical chest compression device with decompression to 2 cm above the resting level of the chest resulted in higher cardiac output, cerebral and carotid blood flow in addition to a trend towards higher end-diastolic CPP compared to standard mechanical chest compressions.

## Abbreviations

ABG: Arterial blood gas
ACD-CPR: Active compression-decompression CPR
CO: Cardiac output
CPP: Coronary perfusion pressure
CPR: Cardiopulmonary resuscitation
CVBG: Central venous blood gas
DAP: Diastolic aortic pressure
DRAP: Diastolic right atrial pressure
ETCO_2_: Expired end tidal carbon dioxide
ITD: Impedance threshold device
MAP: Mean aortic pressure
MRAP: Mean right atrial pressure
SAP: Systolic aortic pressure
SD: Standard deviation
SRAP: Systolic right atrial pressure
VF: Ventricular fibrillation
